# Design of Blended Teaching Model Based on Emotion Recognition and Language Learning

**DOI:** 10.3389/fpsyg.2022.917517

**Published:** 2022-07-29

**Authors:** Xuelian He, Zhenhuan Liu

**Affiliations:** ^1^College of Chinese Language and Literature, Jinan University, Guangzhou, China; ^2^Chong Qing Preschool Education College, Chongqing, China; ^3^School of International Education, Philippine Christian University, Manila, Philippines

**Keywords:** emotion recognition, language learning, blended teaching, teaching model, network platform

## Abstract

With the vigorous development of Internet technology, great changes have taken place in all aspects of human society. This change is also having an increasingly significant impact on the education sector. It is even a trend to subvert the tradition. This also makes the student's identity a passive recipient of knowledge. At the same time, the final orientation of China's education model is the result of examinations, and there is little guidance for students' interest. Of course, traditional teaching also has the ability to enable students' subject knowledge to be systematically established, and to communicate with teachers in the teaching process, which can improve students' learning efficiency in the classroom. In the face of the information explosion in today's society and the rapid development of the above-mentioned technologies, the change of students' learning mode of knowledge has also ushered in an opportunity to change. The purpose of this paper is to study the establishment of a blended teaching model that combines traditional classrooms with network applications. With the help of the characteristics of network big data, it transforms students' passive learning identities, and combines offline traditional learning classrooms with online learning. In addition to the advantages mentioned above, the advantage of online learning is that some network science and technology can be used for the online learning platform to serve the entire teaching process. Therefore, this paper proposes a blended teaching model based on the network platform for students' emotion recognition and language learning result analysis. And from the experimental results in this paper, it can be seen that the recognition evaluation rate of HTMC, the feature of emotion recognition, is 71.52% and the recognition frequency of ETMC is 73.89%. The above two recognition parameters can better reflect the emotional changes in the mixed teaching process.

## Introduction

Today's society is an era of information explosion and technology explosion. The two are mutually reinforcing, and they are making great changes in our lives, including in the important field of education, of course. Because the above factors have changed the way people acquire knowledge, and with the development of network science and technology, a large amount of knowledge can be quickly and conveniently obtained by people. In addition, the characteristics of the Internet's transmission of data also make it possible for systematic learning outside the classroom. Because this feature requires less time and space than traditional classrooms. At the same time, the forms of online classes can also be diverse, which has a better effect on cultivating students' autonomous learning than traditional classes, and can promote students' creative thinking. Of course, pure online teaching will also make it difficult for the purpose of online teaching to achieve what it should be due to the separation between teachers and students and the inability to achieve interaction like traditional classrooms. Of course, pure online teaching will also make it difficult for the purpose of online teaching to achieve what it should be due to the separation between teachers and students and the inability to achieve interaction like traditional classrooms.

This paper explores the blended teaching model based on emotion recognition and language learning. The online learning part of blended teaching is realized through the Internet. For this feature, this paper will build an online learning platform with emotion recognition and language learning analysis capabilities, so that the data collection of students' own changes can be realized in the process of online learning. Then through the relevant data processing and analysis, the obtained student information is analyzed accordingly. Based on the feedback of the obtained results, the blended teaching mode is designed and adjusted. In this way, it can solve the defects such as the inability of teachers and students to interact in online teaching and the lack of learning feedback in traditional face-to-face classrooms.

For the research on blended teaching methods, there are different research angles, such as the combination of traditional classroom and online classroom. Ndayisenga et al. (2021) used the framework of Arksey and O'Malley to study the blended teaching model. The result would be improved educational outcomes. Gupta and Sharma (2021) found that online teaching for students has the advantages of anytime, anywhere access, saving time, promoting learning retention, and reducing commute/transportation costs. HV Tran and his team believed that research findings on blended teaching models provide evidence for the implementation and development of a systematic style-based blended teaching model.

It has an effective role in improving the teaching performance of teachers and helps to improve the teaching quality of higher education (Tran et al., 2021). Ko et al. (2021) aimed to provide insights on bottom-up transformation in teacher-initiated blended teaching. In this way, teachers can realize the transformation of the new teaching mode. Starting from the introduction of China's minority education, Lu and Price (2018) discussed the current status of English education in China's minority areas. This is the point of view for minorities. Wang and Wang (2017) proved that the basic functions of the designed online teaching platform are achievable. His point of view is somewhat related to the research angle of this paper. Adnan et al. (2017) detailed the results of a survey of expectations and satisfaction with a multinational teacher development program for online teaching. GoaCodrua recommended careful study of specific learning/teaching strategies and mapping of any changes in attitudes or practices of teaching/learning in digital formats for specific groups (Goşa and Mureşan, 2021). This research perspective provides a new way for hybrid perspectives. The different studies on the blended teaching model above provide new content filling for the research content of the blended teaching model. However, most of the above studies are biased toward the research on the form of blended teaching, and have not deeply explored the transformation between blended teaching and traditional teaching models. At the same time, the feedback model for blended teaching is not complete enough.

The innovations of this paper are as follows: (1) The combination of traditional classroom and online classroom makes the advantages of the two complement each other, and the shortcomings of the two can also complement each other, and relevant researches have been carried out on their respective advantages and disadvantages. (2) Use the online learning platform of blended teaching to analyze students' emotional responses and language learning, and apply the feedback to the design of blended teaching. (3) This paper makes a more in-depth study on the application of network science and technology in blended teaching, and provides an intermediary method for the blended teaching mode of traditional classroom and online classroom.

## Establishment Method of Blended Teaching Mode Based on Emotion Recognition and Language Learning

### Emotion Recognition System From Multiple Perspectives

As the highest primate on earth, the expression of emotion is one of the characteristics of human beings. Through the recognition of human emotions, a large amount of information can be obtained and an in-depth understanding of the individual can be enhanced. This article is an analysis of emotion recognition and language learning, that is, an in-depth understanding of students, in order to design a teaching model. Recognizing emotion from multiple perspectives has also become a necessary research topic (Rodic and Rodic, 2020).

#### Multi-Angle Emotion Recognition Method

**Figure 1:**
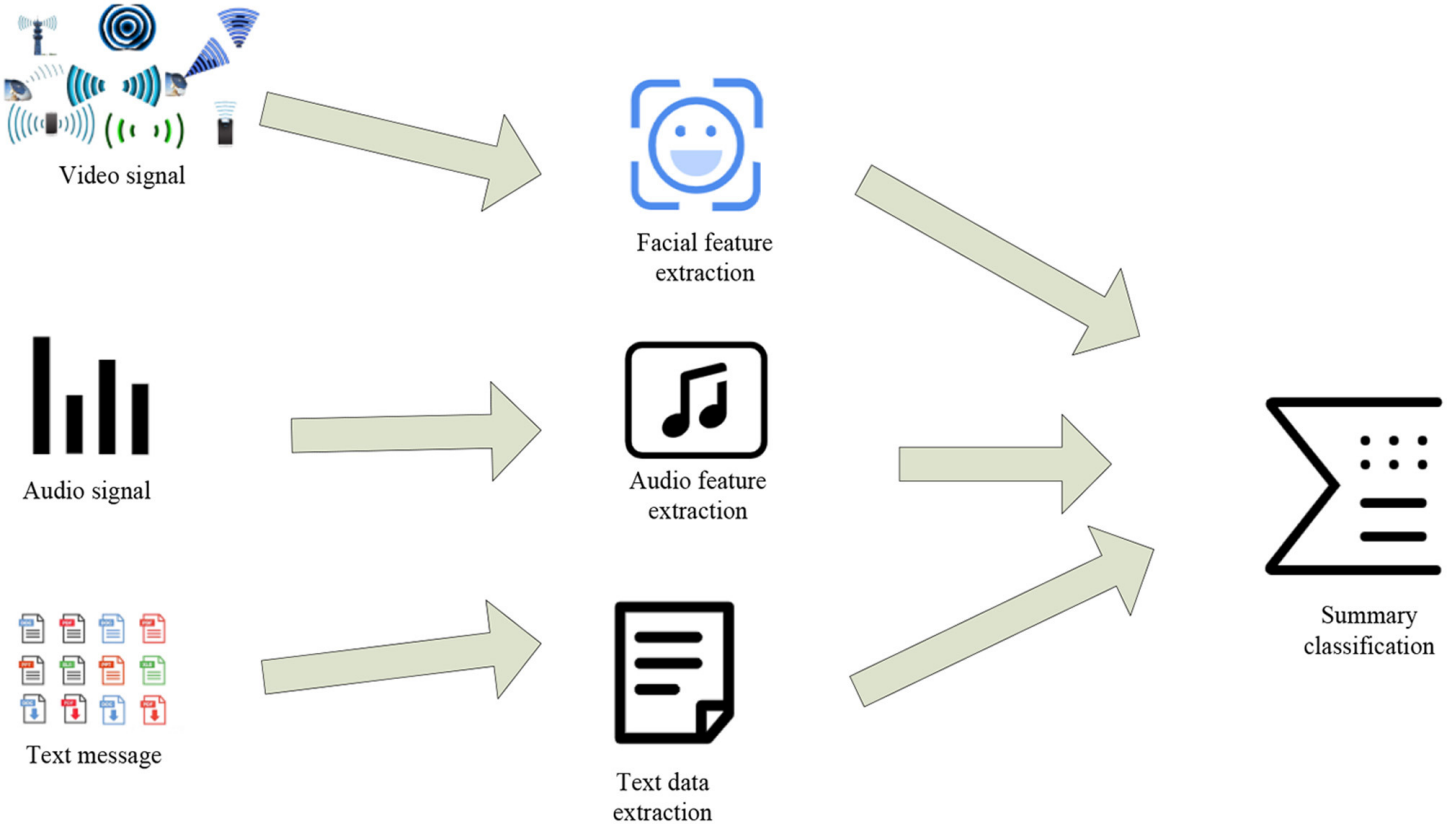
Illustration of multi-angle emotion recognition extraction.

There are many ways to realize multi-angle emotion recognition, which are mainly divided into three types, namely rule-based fusion method, classification-based fusion method and deep learning-based fusion method. The practical application of multi-angle emotion recognition is of great benefit to the hybrid teaching mode, but some related technologies need to make corresponding breakthroughs to achieve it. The following is a brief introduction to the combined system (Rhode et al., 2017). The following is the perspective for extracting emotion recognition features from multiple perspectives, as shown in Figure 1.

**Figure 2:**
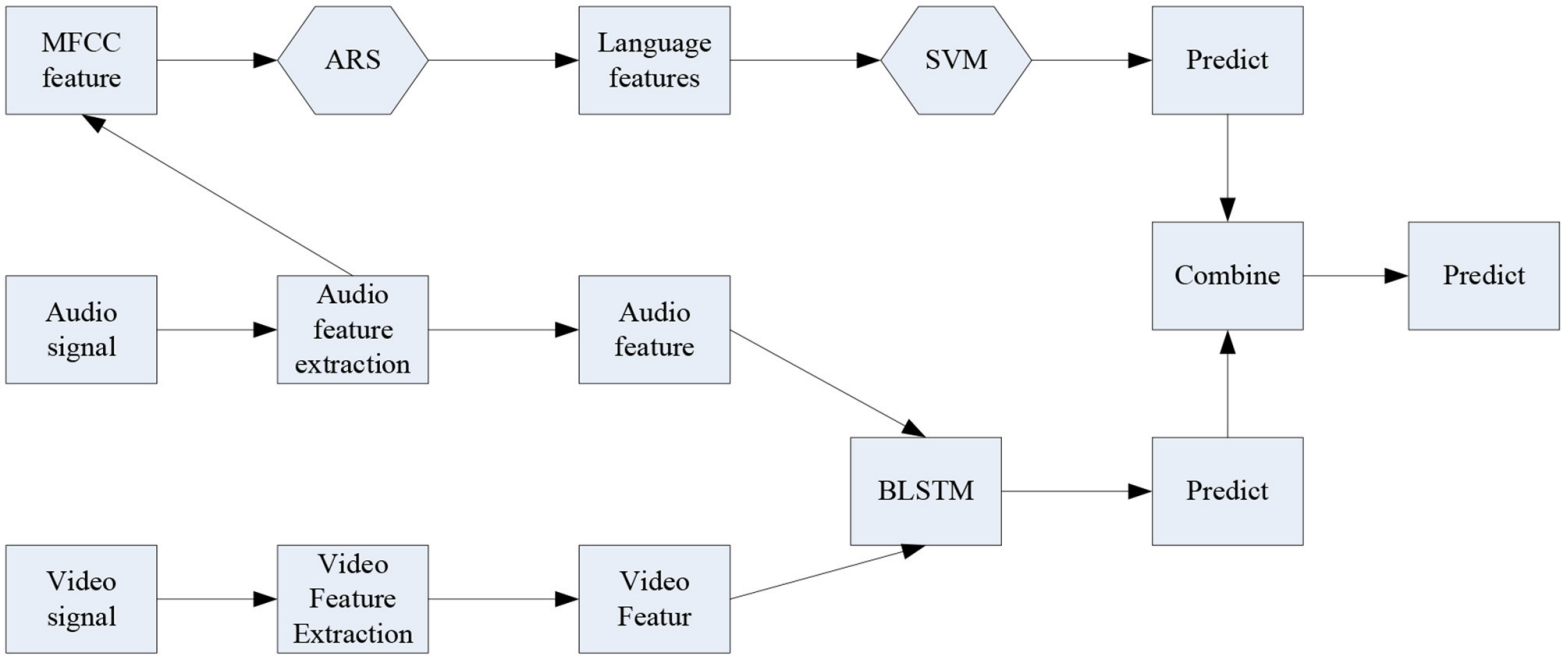
Multi-angle combined information processing flow figure.

Figure 1 shows three perspectives of emotion recognition, which can realize multi-faceted and multi-level reading and recognition of human emotions. The relationship between the three angles is very close. Although the entry points of the above three angles are different, they are all based on the recognition of the person themselves, so it is necessary to apply the corresponding technology to combine the above three angles to serve the design of the final teaching model (Chris, 2017). The combined information processing flow is shown in Figure 2.

The realization of the multi-angle emotion recognition method is inseparable from the interconnection between the algorithms of each part. There are three specific methods of its combination. Through the use of appropriate methods, the establishment of the emotion recognition system can be made more complete. This also contributes to a more comprehensive understanding of students in the teaching process (Javid et al., 2021).

#### Multi-Angle Emotion Recognition Combined Algorithm

**Figure 3:**
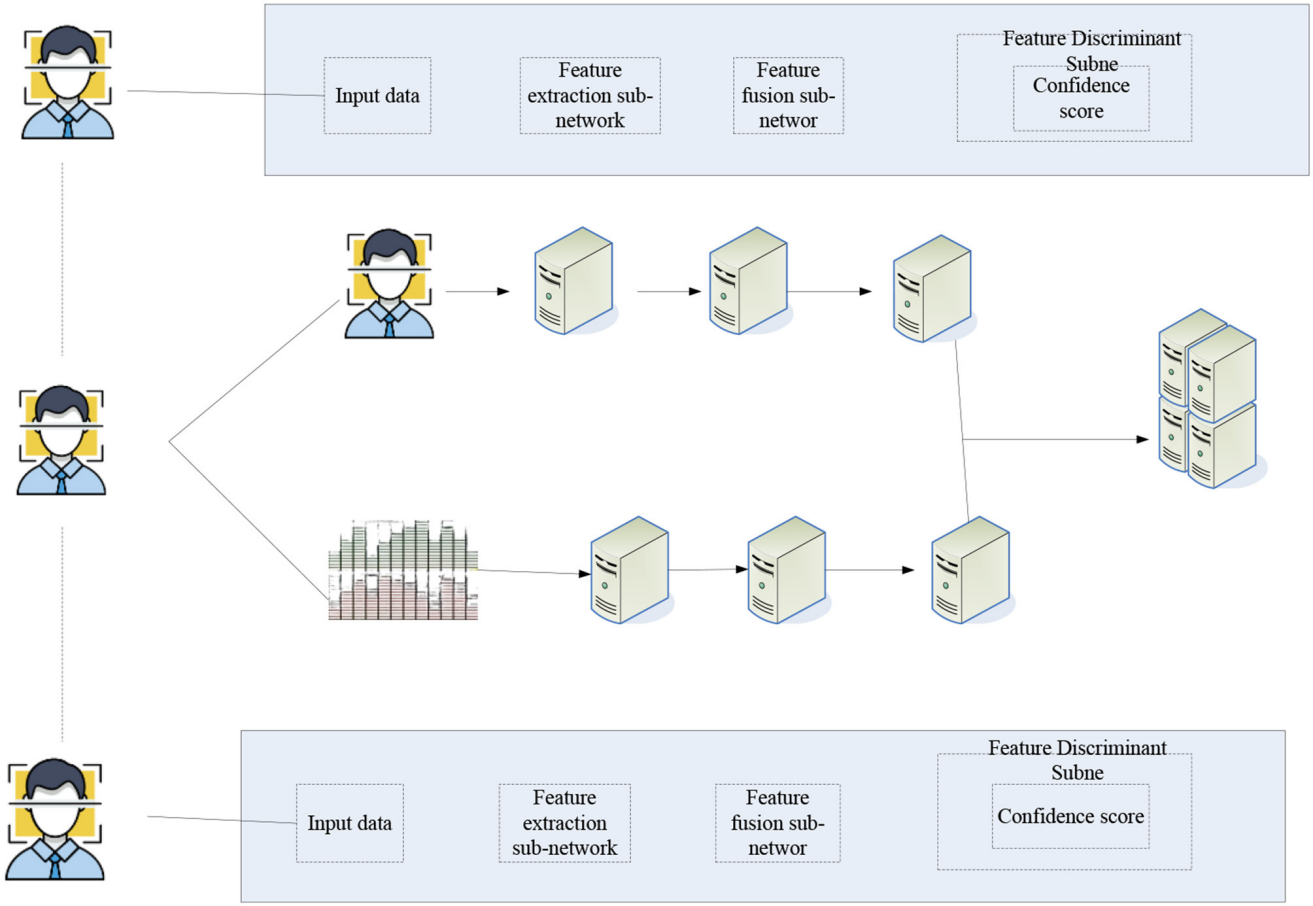
Algorithm structure diagram of multi-angle combination.

The algorithm proposed here is mainly to solve the problem of relative independence faced by the combination of multiple angles. The overall architecture of the algorithm is shown in Figure 3.

Figure 3 is an overall combined frame diagram. For key frame pictures, first locate the eyes of the person, then measure the proportion of the face through the pupil distance of the eyes, and then use face recognition to determine the bounding box of the face. Finally, the face is normalized according to the interpupillary distance, and the facial expression data map is obtained. For audio data, first convert the signal from the time domain to the frequency domain by calculating the mel inverse frequency of the audio. In order to obtain more dynamic characteristics of sound, the first-order and second-order difference calculations are performed on the spectrogram, and the two-dimensional signal is further expanded into a three-dimensional signal to obtain a human voice emotional feature map. First of all, the problem of combining audio and video needs to be solved. Solving the main problem of combining audio and video is to analyze the presence of picture signals and the presence of speech signals. The information carrier of both is a set of continuous sequence and signal combined data. However, the information sampling rates of the two are different, so images and audio need to be processed differently (Liu H. et al., 2021).

#### Processing Methods for Video and Audio

The processing of video and audio information data, that is, the processing of video streams, includes slice-cutting of continuous sequence signals, and correlation processing of image transmission data and audio data, respectively. For the first part, that is, the processing of the video stream, the superimposed dynamic cutting method is adopted in this paper. The processing method for the picture information data is to cut the face of the person along the boundary of the face in the picture, and then use the distance between the pupils to perform the corresponding processing, which can ensure the size of the picture and the integrity of the content (Bulut and Del, 2021). The last is the processing of audio data in the video stream. The first step is to two-dimensionalize the data. Here, the classical algorithm STFT is used for analysis and processing. When processing audio data, the high frequency part of the audio should be expanded first, and some information in the sound should be obviously processed, which can effectively retain the effective information of the sound. The amplification of audio can be expressed by the following formula:


(1) $$\begin{array}{l} U\;(x)=1-\frac{\varepsilon }{x}. \end{array}$$

The value range of ε in the above formula is 0.88–1. After the above process of data 2Dization, the next step is to 3Dize the data, which can be expressed by the following formula:


(2) $$\begin{array}{l} \Delta_{t}=\{\begin{array}{cc} L_{t+1}-L_{t},t<M & \;\\ \; & \frac{\overset{M}{\underset{m=1}{\sum }}m\;(L_{t+m}-L_{t-m})}{\sqrt{2\overset{M}{\underset{m=1}{\sum }}m^{2}}}\\ \; & M_{t}-M_{t-1},t\geq P-M \end{array} \end{array}$$

Δ_*t*_ of the above formula is the difference value of the first picture, and Q represents the number of pictures. The end result is a graph that can represent the emotion of the sound (Liu P. et al., 2021).

### Establishment Method of Facial Emotion Recognition System

Blended teaching is a new teaching mode that combines offline face-to-face teaching and online network teaching. This paper introduces the method of emotion recognition into blended teaching from the network teaching part of online teaching. The operation of this method is based on the combination of Internet science and technology, and the use of Internet-related technologies to establish a computer system with emotion recognition for the online platform of blended teaching. The system can have the ability to recognize the user's emotion, and can make corresponding feedback on the recognized content. The establishment of its entire system is the result of the fusion of various technical disciplines (Fitriani et al., 2021). Emotion recognition integrates digital image processing, speech signal processing, pattern recognition, psychology and other disciplines.

#### Introduction to the Process of Facial Emotion Recognition

**Figure 4:**
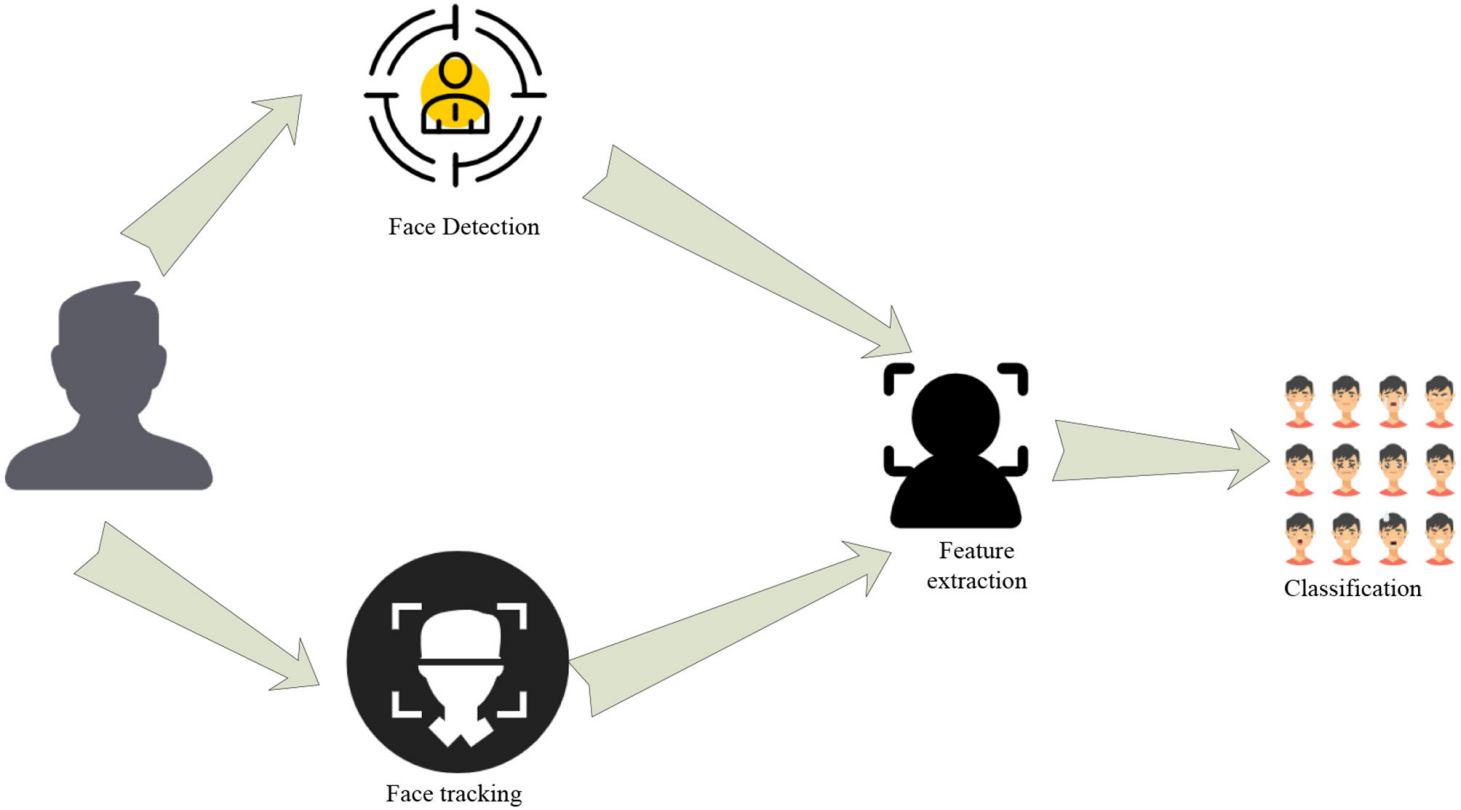
Flowchart of facial emotion recognition.

There are many ways to express human emotions. The first is to introduce the emotion recognition technology of human face and related principles. Figure 4 below is an introduction to the process of facial emotion recognition:

Face emotion recognition can be achieved by deep learning algorithms in the current environment. The process features are the same as the description in Figure 4, that is, it includes the preprocessing of the image of the human face, the extraction of facial emotion features, and finally the classification of the recognized expressions until the final output result (Sato and Chen, 2021). With the vigorous development of deep learning and the increasing maturity of artificial intelligence-related technologies, the implementation of the above process is simpler than previous technologies.

##### Facial Emotion Recognition Extraction Method

For the realization of facial emotion recognition technology, the most important thing is the ability to extract the features of the human face that can best represent the emotion of the person at that time. The previous realization of facial emotion recognition was based on the use of various algorithms, but the use of different algorithms will produce large differences in the results of facial emotion recognition. Because of the differences between the various algorithms and the idealization of the data during operation, they are different from the actual situation. First, the Gabor algorithm is introduced (Zhanq et al., 2021), which is a linear filter in computer image processing. The use of this algorithm is also generally related to the processing of pictures. Its function algorithm for image feature extraction can be expressed by the following formula:


(3) $$\begin{array}{l} \lambda \left(x,y,z,w\right)=\frac{1}{2\pi \varepsilon^{2}}a^{-\left(\frac{x_{1}^{2}+y_{1}^{2}}{2\varepsilon^{2}}\right)}a^{jzx_{1}} \end{array}$$

(x, y) in the above formula represents the specific position of the pixel when the image is recognized, and ε is the standard value of the deviation in the horizontal and vertical directions of the table x and y. The following are the relevant formulas for x and y, respectively:


(4) $$\begin{array}{l} \{\begin{array}{c} x_{1}=x\cos w+y\sin w\\ y_{1}=y\sin w+y\cos w \end{array} \end{array}$$

The above formula defines x and y, respectively, and the image of the filter can be expressed by the above formula. The mathematical formula of the complex number function that can be finally obtained is as follows:


(5) $$\begin{array}{r} f\;(x,y,s,a,c,m,n)=\exp\left(-\frac{{x_{1}}^{2}+n^{2}{y_{1}}^{2}}{2\varepsilon^{2}}\right)\\ \exp\left(j\;(2\pi \frac{x_{1}}{a}+q)\right). \end{array}$$

The real part of the function can be expressed by the following formula:


(6) $$\begin{array}{l} f\;(x,y,s,a,c,m,n)=\exp\left(-\frac{{x_{1}}^{2}+n^{2}{y_{1}}^{2}}{2\varepsilon^{2}}\right)\cos\left(2\pi \frac{x_{1}}{a}+q\right). \end{array}$$

In addition, there is the imaginary part of the function, and its expression formula is as follows:


(7) $$\begin{array}{l} f\;(x,y,s,a,c,m,n)=\exp\left(-\frac{{x_{1}}^{2}+n^{2}{y_{1}}^{2}}{2\varepsilon^{2}}\right)\sin\left(2\pi \frac{x_{1}}{a}+q\right). \end{array}$$

In the above formula expression, s represents the sine wavelength function, a represents the direction of the algorithm, and n is the ratio between height and width in space, the symbol q represents the characteristic representation of the filter function (Ullah et al., 2021).

In addition, for the implementation of facial emotion recognition, the ability to collect and analyze local features is also required. Local binary descriptors (lbp) are introduced here. Its feature is that it can better identify the directionality of the picture, and the application of this method improves the computational efficiency (Khtere and Yousef, 2021). Finally, the spatial structure formula of lbp can be obtained:


(8) $$\begin{array}{l} lbp\;(x,y)={\overset{x}{\underset{j=0}{\sum }}m\;(p_{j}-p_{a})}^{2^{j}} \end{array}$$

After applying lbp to process the collected facial emotion images, the facial features can be described by computation. The specific feature image information includes the edge of the image and the feature points of the image itself.

##### Active Recognition Shape Model for Facial Emotion Recognition

To read the shape features of the face, it is necessary to accurately determine the position of the face, and the final result of the extraction needs to match the actual face features as much as possible. The model that can actively recognize the shape adopted in this paper is one of the better ways to solve this problem (Azrva et al., 2021). The basis of the method model is to read the face that has marked the feature points. The features of the face can be expressed by the following formula:


(9) $$\begin{array}{l} P=\left\{\left(L_{a},M_{a}\right)\;a=1,...,n;M_{k}={\left(w_{t}^{r},z_{t}^{r},y_{t}^{r}\right)}^{T}\right\} \end{array}$$

In the above formula, n represents the total number of experimental subjects tested by this model, and r is the number of feature points marked on the face. Then use the corresponding shape algorithm on the above data, and finally people can get the following formula model:


(10) $$\begin{array}{l} F=\bar{F}+Q_{F}B_{F}. \end{array}$$

In the above formula, $\bar{F}$ represents the average value of the shape data of the experimental object, and *B*_*F*_ represents the parameters of the above algorithm model. After obtaining the corresponding punctuation model of the facial features, it is necessary to effectively describe the features of each feature point. Therefore, this paper introduces the processing model of feature point characteristics. Its related expression formula is as follows:


(11) $$\begin{array}{l} f\;(L_{a})={\left(L_{a}-\bar{L_{b}}\right)}^{x}\overset{-1}{\underset{b}{\sum }}\left(L_{a}-\bar{L_{b}}\right) \end{array}$$

The establishment of the above model is a method to reduce the similarity of feature points. The use of this method makes the results of the previous shape recognition model more complete. At the same time, since the expression of emotion by human face, in addition to the emotion with a greater degree of discrimination, the recognition of relatively similar emotion will also increase the difficulty of recognition.

### Relevant Algorithms and System Establishment of Language Emotion Recognition

Speech emotion recognition has a certain correlation with the facial emotion recognition above, and also has all its own uniqueness. The characteristics of speech emotion recognition include the same emotional information as facial emotion recognition, and speech emotion recognition also has the characteristics of conveying semantic information (Jayathilakan, 2021). Through the recognition of speech emotion, students' emotional state can be inferred, and then the result can be applied to the final blended instructional design.

#### Basic Theory of Language Emotion Recognition

Before the emotion recognition of language, the following problems need to be solved. The first is to properly classify the existing emotions, and then it is necessary to establish a language emotion recognition database before identifying them. Next is the extraction of language emotion features and the establishment of the final language emotion recognition model and the elaboration of the algorithm. The first is to introduce the method model of sentiment classification. The model used in this paper is a three-dimensional emotional model also known as the PAD model. The model expresses different emotions through three dimensions. Its first two dimensions, dimension pleasure, refer to the valence dimension, which assesses whether an affective state is positive or negative. The dimension Arousal refers to the motivation dimension, which reflects the degree of emotional strength. Its third dimension, dominance, refers to the control dimension, which describes the degree of control of the individual's intentions, whether it is dominant or submissive. The use of the PAD model can better describe human emotions. The first is to establish the emotional space of PAD, and the final result is to generate a simplified scale of PAD (Gupta and Sharma, 2021), as shown in Table 1.

**Table 1:** PAD affect scale.

**Item number**	**Emotional adjectives (left end)**	**Annotation level**	**Emotional adjective (right end)**
V1	Angry	−4, −3, −2, −1, 0, 1, 2, 3, 4	Interested in
V2	Sober	−4, −3, −2, −1, 0, 1, 2, 3, 4	Sleepy
V3	Controlled	−4, −3, −2, −1, 0, 1, 2, 3, 4	Master
V4	Friendly	−4, −3, −2, −1, 0, 1, 2, 3, 4	Contemptuous
V5	Peaceful	−4, −3, −2, −1, 0, 1, 2, 3, 4	Excited
V6	Dominated	−4, −3, −2, −1, 0, 1, 2, 3, 4	Submissive
V7	Painfully	−4, −3, −2, −1, 0, 1, 2, 3, 4	Happy
V8	Interested in	−4, −3, −2, −1, 0, 1, 2, 3, 4	Relaxed
V9	Humble	−4, −3, −2, −1, 0, 1, 2, 3, 4	Proud
V10	Excited	−4, −3, −2, −1, 0, 1, 2, 3, 4	Irritated
V11	Conservative	−4, −3, −2, −1, 0, 1, 2, 3, 4	Surprised
V12	Influential	−4, −3, −2, −1, 0, 1, 2, 3, 4	Affected

The Table 1 shows that V1–V12 are different observation items, −4 to 4 are the scores of each item, and the final result is the target dimension value of emotion. In addition, as the reference value of emotional target value, Table 2 is the three-dimensional emotional value of some basic emotions:

**Table 2:** Some base sentiment PAD values.

**Number**	**Emotion**	***P***	***A***	***D***
1	Happy	2.85	1.25	1.50
2	Boring	−0.64	−1.50	−0.96
3	Sad	−0.92	0.15	−0.69
4	Anger	−2.01	1.02	0.5
5	Surprise	1.88	1.85	0.35

Table 2 shows the five basic emotional PAD values, which are derived from the joint analysis of the three-dimensional emotional model and the simplified PAD scale, respectively.

#### Feature Extraction of Language Emotion Recognition

**Figure 5:**
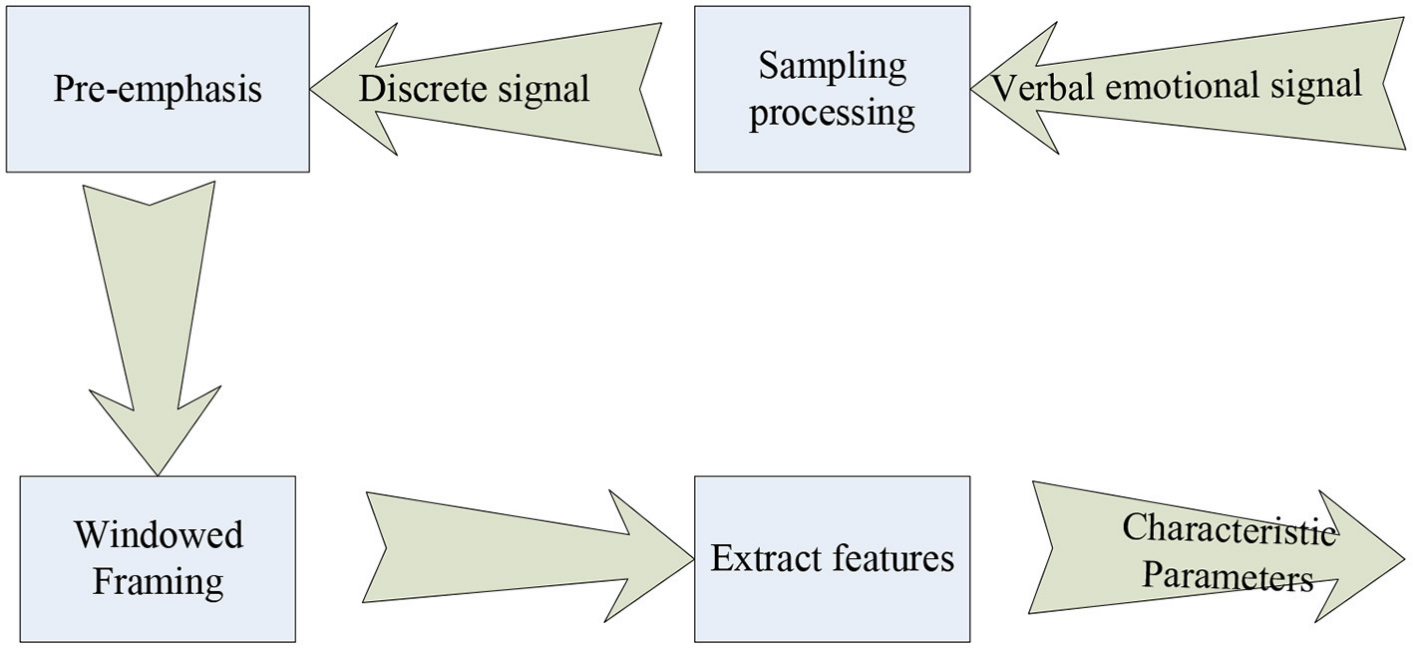
Flowchart of language emotion recognition.

The extraction of language emotional features is different from the extraction of facial emotional features, because the feature characteristics they are based on are completely different. The characteristics of sound include rhythmic characteristics including energy, fundamental frequency, etc., timbre characteristics represented by formants, and classical spectral characteristics. The characteristics of language are mainly the timbre of the human voice and the rhythm of the voice. The following is the specific process of language emotion recognition (Niculescu and Dragomir, 2021), as shown in Figure 5.

The process of language feature extraction is shown in Figure 5. The first is to process the language signal, and finally the extraction of language emotion parameters can be realized. The first thing to extract is the rhythm feature of the sound. This feature is second only to the language quality in terms of language features, and it mainly describes the pitch of the language and other characteristics of the sound. The rhythm of the sound itself also has several characteristics, which will be introduced one by one below. The first is to describe the energy characteristics of language rhythm characteristics. Although the energy of language changes with time, its energy is relatively stable in a short time interval. The energy extraction algorithm can be expressed by the following formula:


(12) $$\begin{array}{l} F_{M}=\overset{+\infty }{\underset{N=-\infty }{\sum }}{\left[z\;(N)\;r\;(M-N)\right]}^{2}=\overset{M}{\underset{N=M-\left(N-1\right)}{\sum }}{\left[z\;(N)\;r\;(M-N)\right]}^{2} \end{array}$$

The above formula is the expression of language sound energy in a short time interval. The emotional language signal is x(n) after sampling, the window function is w(n), and the window length is N. Because when the sound energy is stable, its energy is also out of the steady state. And due to the difference of people's emotions, it is usually manifested in the level of the volume when speaking, that is, the difference in energy.

Next is a discussion on the fundamental sound frequency of sound. For the acquisition of this frequency, the Z periodic vibration of the human voice can be described. Its expression formula is as follows:


(13) $$\begin{array}{l} T_{i}\left(a\right)=\overset{+\infty }{\underset{N=-\infty }{\sum }}z\;(N)\;r\;(M-N)\;Z\;(N+a)\;z\;(M-a-N). \end{array}$$

The above formula is the self-correlation algorithm of the time period, among them, *T*_*i*_*(a)* is the functional operation on the emotional signal of the language.

Then there is the observation of each frame of the signal over a short period of time to see how many times it passes through the zero value. Since speech has stable characteristics in a short period of time, the definition of the above times can be defined as follows:


(14) $$\begin{array}{l} Q_{z}=\overset{+\infty }{\underset{N=\infty }{\sum }}|sgn\;[x\;(M)]-sgn\;[x\;(M-1)]|r\;(M-N) \end{array}$$

The sign in the above formula represents the corresponding symbolic function, and its expression is as follows:


(15) $$\begin{array}{l} sgn\;[x\;(M)]=\{\begin{array}{l} -1,x\;(M)<0\\ 0,x\;(M)=0\\ 1,x\;(M)>0 \end{array} \end{array}$$

W(M) represents the window function, and the window length is represented by L, and its expression formula is as follows:


(16) $$\begin{array}{l} w\;(M)=\{\begin{array}{l} \frac{1}{2L},0<M<L-1\\ 0 \end{array} \end{array}$$

For the last feature of language rhythm, the feature of language speed is extracted. When people communicate, the amount of vocabulary that appears per unit time is the speed of language. It can be expressed by the following formula:


(17) $$\begin{array}{l} v=\frac{t}{m} \end{array}$$

In the formula, v represents the speech rate, t represents the time length of the emotional signal of the unit sentence, and m is the number of syllables in the unit sentence. Next is a discussion of the sound spectrum characteristics of language, which are expressions for vocal systems such as vocal cords, which can be expressed by the following formula:


(18) $$\begin{array}{l} P\;(x)=2595\times \mathrm{lg}\left(1+\frac{x}{600}\right) \end{array}$$

The x of the above formula represents the size of the frequency, and the above formula can only be used for a rough description. It belongs to a non-linear frequency description calculation method.

#### Establishment of Language Emotion Recognition Database

Next is the construction of the database required for language emotion recognition. The construction methods are different, and the corresponding database types are also different. Its construction methods usually include the following: the first is the acquisition of inspiration, the second is the acquisition of inducement, the third is the acquisition of interception and emotional acquisition in the form of performance. The excerpt type refers to the interception from TV, movies and other materials, the emotional expression is directly generated by psychological activities, the emotion is richer, the authenticity is higher, and the content of the dialogue is related to the context. This article will take a guided acquisition approach. This method needs to be implemented by means of audio recording. During the operation, 10 people were allowed to participate and divided into two groups. Each person in each group made 10 language recordings. The results are shown in Table 3.

**Table 3:** Linguistic sentiment database.

	**Male**					**Female**					**SUM**
	**1**	**2**	**3**	**4**	**5**	**6**	**7**	**8**	**9**	**10**	
Happy	10	10	10	10	10	10	10	10	10	10	100
Anger	10	10	10	10	10	10	10	10	10	10	100
Sad	10	10	10	10	10	10	10	10	10	10	100
Startled	10	10	10	10	10	10	10	10	10	10	100
Neutral	10	10	10	10	10	10	10	10	10	10	100
Total	50	50	50	50	50	50	50	50	50	50	500

Table 3 records the five emotions, respectively, and the final collection of language emotion data can only be entered after the research operator judges it to pass. Next is the construction of the language package emotion recognition database, the corresponding data of the six people selected here. The finishing results are shown in Table 4.

**Table 4:** Language emotion recognition database.

	**Male**			**Female**			**SUM**
	**1**	**2**	**3**	**7**	**8**	**9**	
Happy	10	10	10	10	10	10	60
Anger	10	10	10	10	10	10	60
Sad	10	10	10	10	10	10	60
Startled	10	10	10	10	10	10	60
Neutral	10	10	10	10	10	10	60
Total	10	10	10	10	10	10	60
Happy	50	50	50	50	50	50	300

Table 4 shows that 60 valid recordings of each emotion are selected and recorded, and the obtained data will be trained below. Because the speech emotion database is large, the storage of its data needs to be operated according to a certain name, and then the subsequent corresponding recognition can be realized. The specific storage naming method is shown in Table 5.

**Table 5:** Recording storage naming method.

**Emotion category**	**Emotional logo**	**Language identification**		**Gender marker**		**Speaker identification**	**Statement number**	
Happy	H	C	E	M	F	0–9	T000-499	R000-299
Anger	A	C	E	M	F	0–9	T000-499	R000-299
Surprise	S	C	E	M	F	0–9	T000-499	R000-299
Sad	G	C	E	M	F	0–9	T000-499	R000-299
Neutral	N	C	E	M	F	0–9	T000-499	R000-299

##### Establishment of Language Emotion Recognition Model

**Figure 6:**
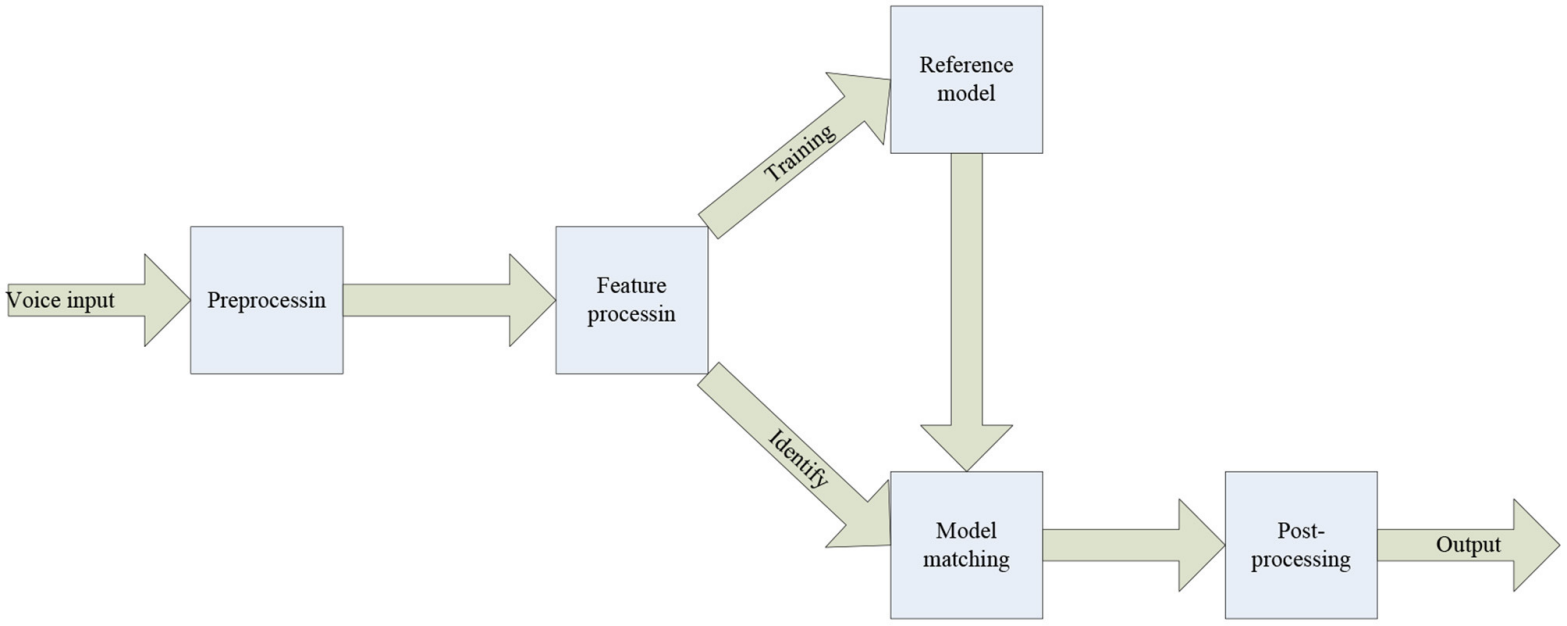
Linguistic emotion recognition model.

Through the establishment of the above database and the extraction of language feature data, the next step is to establish the language emotion recognition system, and its model structure is shown in Figure 6.

Figure 6 is a general overview for linguistic emotion recognition. Language emotion recognition can be divided into three steps: preprocessing, extraction of speech emotion features, and emotion recognition. The initial part of the system is the pre-emphasis of speech signals. The purpose of this is to expand the frequency coverage of the signal, and then it can be more convenient to analyze the parameters of the sound. The calculation method for pre-emphasis is shown in the following formula:


(19) $$\begin{array}{l} W\;(x)=1-\beta x^{-1} \end{array}$$

**Figure 7:**
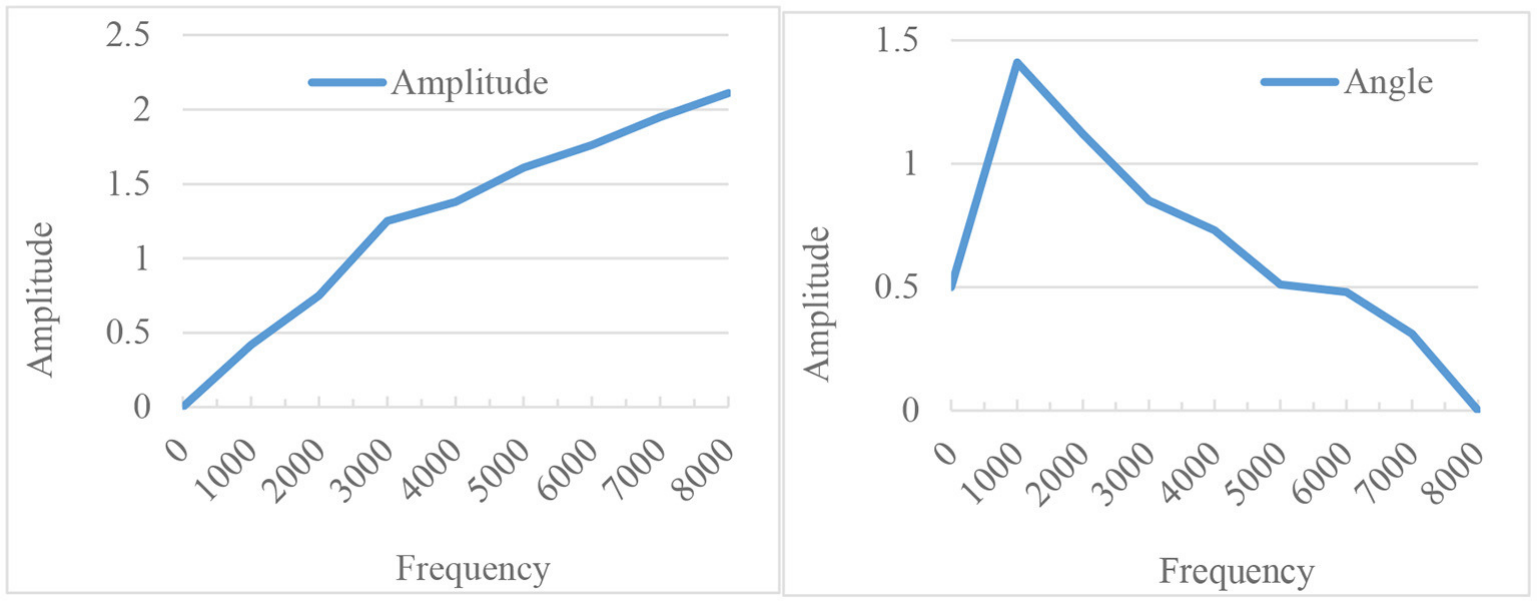
Filter frequency spectrum diagram.

The β in the above formula is the coefficient value of the pre-emphasis processing formula, and the range is between 0.89 and 1. Since pre-emphasis can only be achieved through a filter, the frequency spectrum characteristics of the filter are shown in Figure 7.

The next step is to correlate the uneven part of the sound signal, and here are two processing algorithm modes. The first is the Hamming window calculation method:


(20) $$\begin{array}{l} p\;(x)=\{\begin{array}{l} 0.56-0.46\cos\left[\frac{2\pi x}{\left(M-1\right)}\right]\\ 0 \end{array},0\leq x\leq M-1. \end{array}$$

At the end of the above expression formula, the signal after windowing the language information can be obtained, and then the Hanning window algorithm, such as the formula:


(21) $$\begin{array}{l} p\;(x)=\{\begin{array}{l} 0.5\left(1-\cos\left(2\pi x/\left(M-1\right)\right)\right),0\leq x\leq M-1\\ 0 \end{array} \end{array}$$

The formulas are the establishment of the language emotion recognition system and the elaboration of the related algorithms.

### Blended Instructional Design Method Based on Emotion Recognition

**Figure 8:**
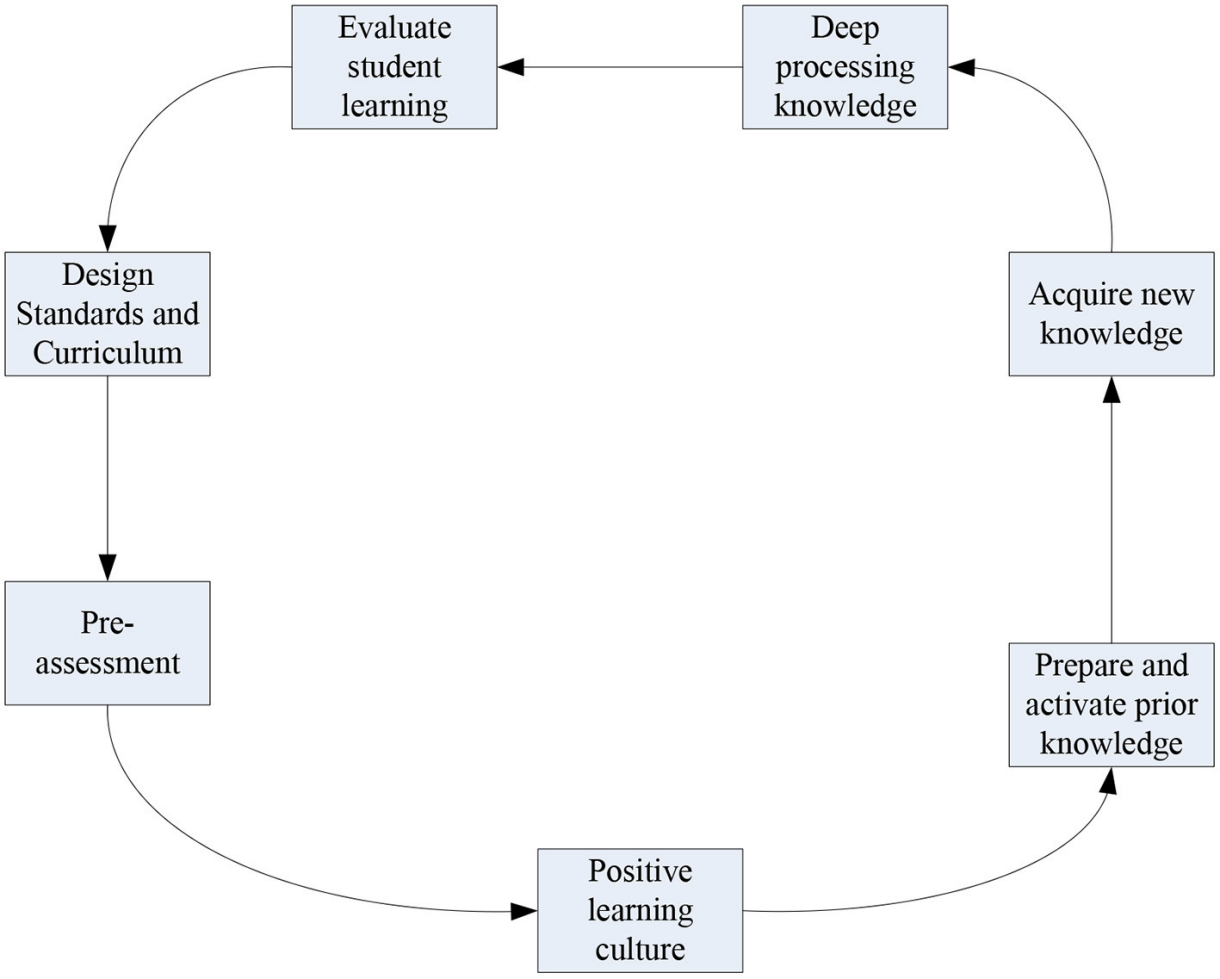
Blended teaching learning model diagram.

For the establishment of a blended teaching method based on emotion recognition, we first need to know some design principles. The first is to be teacher-led and to treat students as the main body of the blended teaching process. Let teachers get rid of the traditional role of knowledge instillers as much as possible, so as to better encourage students. Then, in the process of blended teaching, questions need to be used as the orientation of the curriculum. Through the establishment of the above-mentioned emotion recognition system, teachers can use the system to discover and give timely feedback to students' problems. Finally, it is necessary to use both offline and online teaching modes to improve students' higher-level ability and literacy, and pay scientific attention to the entire learning process through the emotion recognition system. Courses are adjusted accordingly through systematic feedback and information processing. Blended teaching is designed to promote better learning for students. The process figure is shown in Figure 8.

The introduction to the blended teaching process is as in the above section, and the process in Figure 8 reflects the flow of students learning through the blended teaching model. Three stages from front-end analysis, teaching resource and activity design, and teaching evaluation design. For the design of blended teaching, it is necessary to do a good job in the analysis before teaching, and through the analysis and processing of the results of emotion recognition, in order to lay the foundation for further learning.

## Experiments and Results of the Blended Teaching Model Based on Emotion Recognition

### Experiments and Results Based on Facial Emotion Recognition

This paper is to conduct an experimental study on the online teaching part of the blended teaching process. During online learning, teachers and students use the medium of video to achieve teaching. The following is an experimental study on several important facial expressions for facial emotion recognition. The object of its study is the expression in a static state.

**Figure 9:**
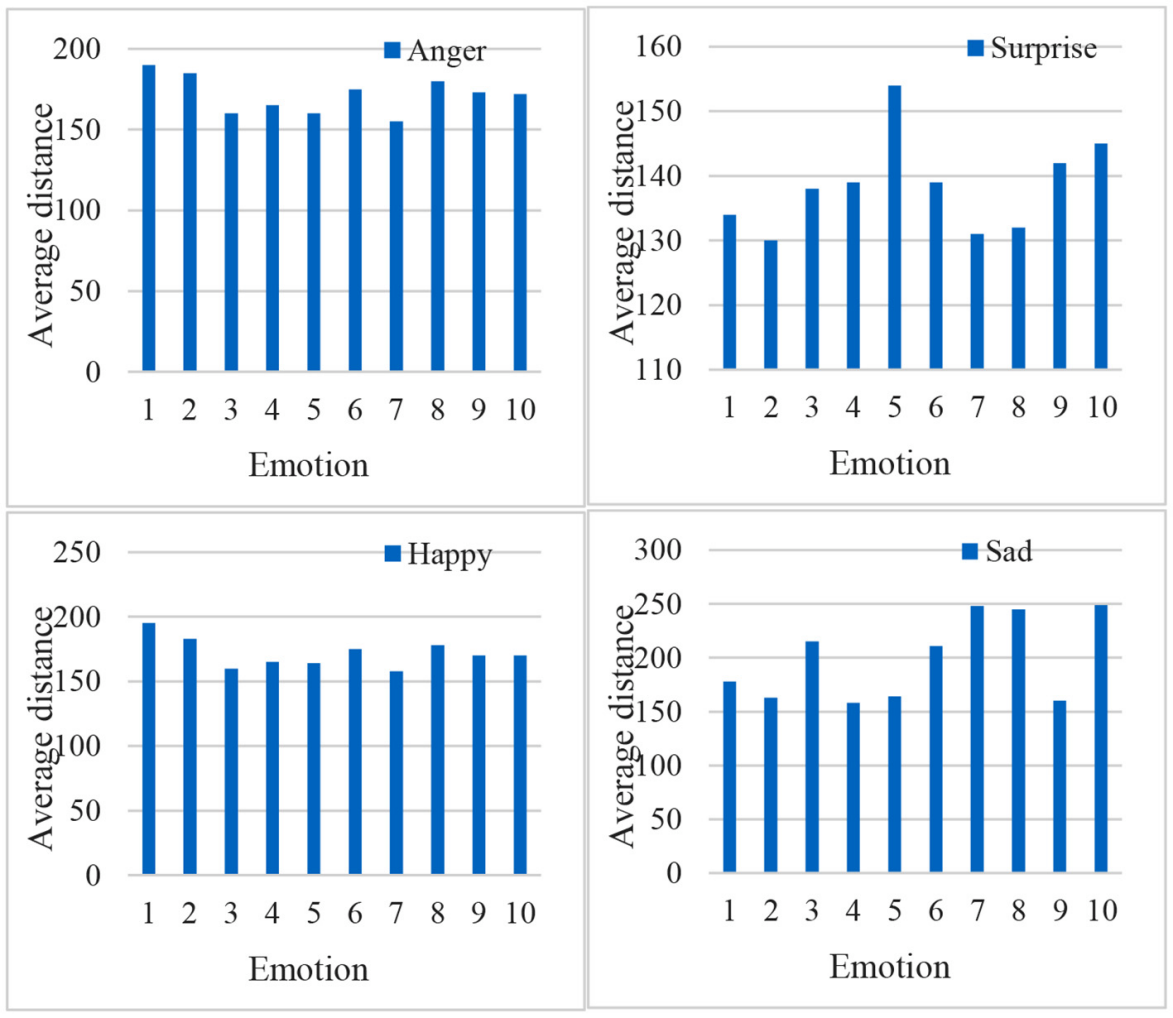
Average pixel distance extraction map for four emotions.

Figure 9 is the result of data extraction from the facial emotion database established above. It is through different emotional expressions for people with different emotional statements. From the results of the above experiments, it can be seen that the facial emotion recognition in the process of online network learning can obtain important image information for students to learn online.

### Experiments and Results Based on Language Emotion Recognition

**Figure 10:**
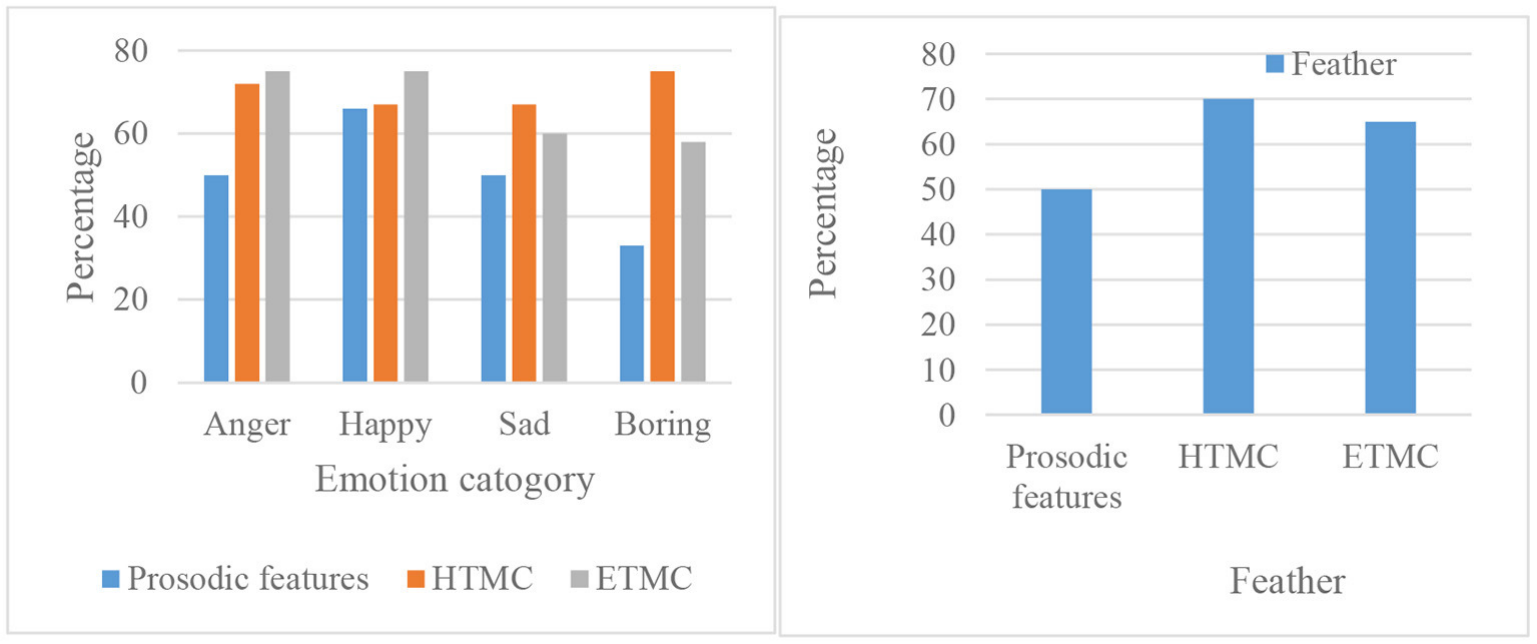
Recognition frequency and average recognition frequency of three features.

The experiments here are carried out on the basis of selecting the language database established above. Because a large amount of corpus information containing emotional features has been entered in the language database, it is necessary to distinguish and identify language emotions first. The results of the processing analysis for the first type of database are shown in Figure 10.

In the left panel of Figure 10, it can be seen that the frequency of recognition of both emotions “anger” and “happy” is more obvious than other emotion forms. The average recognition rate for the recognition of prosody features of sound reaches 50%, and the recognition frequency for ETMC reaches 67.12%. However, the best recognition result in HTMC reaches 71.1%, which is the best performing feature identified among these features.

**Figure 11:**
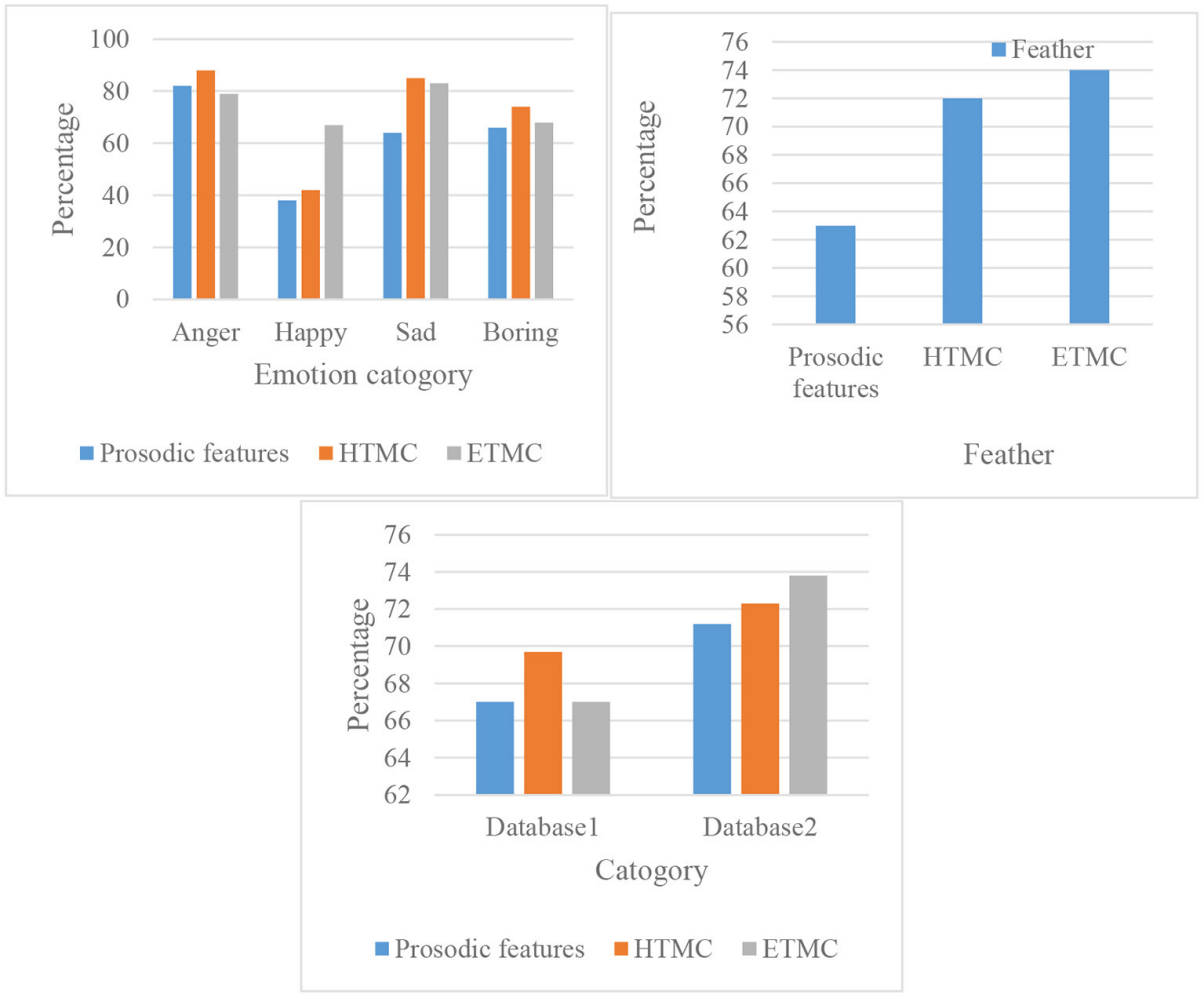
Feature recognition rate and average recognition rate.

For the experimental results in another language sentiment database, as shown in Figure 11. It is also expressed for the recognized frequency and the average of the recognized frequency for the four different emotions.

Figure 11 shows that the average recognition rate of prosodic features is 62.51%, the recognition rate of HTMC is 71.52% and the recognition frequency of ETMC is 73.89%. The parameters of the above three features can be seen that HTMC and ETMC are similar, and the result is the best. For the EMO-DB speech database, the feature ETMC has the highest recognition rate. This shows that among the three spectral features proposed in this paper, the features HTMC and ETMC show better performance, which provides a good foundation for the next feature fusion.

### Blended Teaching Experiment and Results

The status quo of blended teaching can be seen from the literature research, and the blended teaching literature in China has gradually increased. For the research on the form of blended teaching, the results are shown in Table 6.

**Table 6:** Blended teaching keyword dissimilarity matrix.

	**Blended learning**	**Hybrid teaching**	**MOOC**	**SPOC**
Blended learning	0	1	0.9785	0.971
Hybrid teaching	1	0	0.9666	0.9854
MOOC	0.9796	0.9666	0	0.6972
SPOC	0.984	0.9856	0.6970	0

The research on blended teaching in this paper is based on emotion recognition. The advantage is that it can well collect students' learning outcomes and various data in the learning process, give feedback after processing, and then revise the teaching design. The final grades of the experimental class and the control version were compared. Both the experimental class and the control class are taught by the same teacher. The amount and difficulty of the two final exam papers for the experimental class and the control class are equal. The question types of the test paper are prepared according to the requirements of the syllabus, and consist of multiple-choice questions, fill-in-the-blank questions, short answer questions and calculation questions. The ratio of subjective questions to objective questions is 1:1.

## Discussion

This paper is a research on blended instructional design based on emotion recognition. The development of this research is based on the development of contemporary network science and technology and a large number of related disciplines. It is a symbol of the realization of the above technology in the field of education. The research on emotion recognition in this paper first discusses emotion recognition from multiple perspectives, and illustrates the multi-angle emotion recognition through corresponding flowcharts. Then a large number of related algorithms are introduced to build a multi-angle emotion recognition model. This enables teachers to collect students' learning situations from multiple perspectives in the online learning process.

The following is an introduction to the method of facial emotion recognition. For this perspective, the first step is to collect a large amount of facial emotion data to build a database. Then the author uses the Gabor algorithm to calculate the data, so that the influence of small differences between various expressions can finally be reduced. An active recognition shape model is also established, which has a more intelligent role. It enables dynamic recognition of faces in the process of online learning, so as to better improve emotion recognition and pave the way for the final blended teaching. In addition to the recognition of facial emotion, it is also necessary to recognize the emotion of language, which is also one of the important contents of emotion. For the discussion of this part, it first introduces the relevant theoretical concepts, and then collects and extracts language emotion features, which is to prepare for the establishment of language emotion database. Finally, a language emotion recognition model based on the corresponding database is established.

The research on blended teaching can see its development trend from the literature over the years. The research on blended teaching in this paper conforms to the development of the times and combines with the related technologies of emotion recognition, so that it can be discussed.

## Conclusions

Blended teaching is a major trend in contemporary education. Because the development of the times brings a lot of information development, for education and learning, it also needs to be improved in different ways. Because the way of acquiring knowledge has undergone great changes, the article has great practical significance for the research of blended teaching. The application of emotion recognition in blended teaching can be realized through the use of computer technology and a large number of related technologies. However, the addition of the above technologies has made the model of blended education more advanced.

## Data Availability Statement

The original contributions presented in the study are included in the article/supplementary material, further inquiries can be directed to the corresponding author.

## Author Contributions

ZL guiding the research directions and ideas. XH writing and static analysis of data. All authors contributed to the article and approved the submitted version.

## Conflict of Interest

The authors declare that the research was conducted in the absence of any commercial or financial relationships that could be construed as a potential conflict of interest.

## Publisher's Note

All claims expressed in this article are solely those of the authors and do not necessarily represent those of their affiliated organizations, or those of the publisher, the editors and the reviewers. Any product that may be evaluated in this article, or claim that may be made by its manufacturer, is not guaranteed or endorsed by the publisher.
